# Gas chromatography-mass spectrometry analysis, phytochemical screening and antiprotozoal effects of the methanolic *Viola tricolor* and acetonic *Laurus nobilis* extracts

**DOI:** 10.1186/s12906-020-2848-2

**Published:** 2020-03-17

**Authors:** Gaber El-Saber Batiha, Amany Magdy Beshbishy, Luay Alkazmi, Oluyomi Stephen Adeyemi, Eman Nadwa, Eman Rashwan, Amany El-Mleeh, Ikuo Igarashi

**Affiliations:** 1 National Research Center for Protozoan Diseases, Obihiro University of Agriculture and Veterinary Medicine, Nishi 2-13, Inada-cho, Obihiro, Hokkaido 080-8555 Japan; 2 Department of Pharmacology and Therapeutics, Faculty of Veterinary Medicine, Damanhour University, Damanhour, AlBeheira 22511 Egypt; 3 Biology department, Faculty of Applied Sciences, Umm Al-Qura University, Makkah, 21955 Saudi Arabia; 4 Medicinal Biochemistry, Nanomedicine and Toxicology Laboratory, Department of Biochemistry, Landmark University, Omu-Aran, Kwara State 251101 Nigeria; 5 Department of Pharmacology and Therapeutics, College of Medicine, Jouf University, Sakaka, Saudi Arabia; 6 Department of Medical Pharmacology, Faculty of Medicine, Cairo University, Giza, Egypt; 7 Department of Physiology, College of Medicine, Al-Azhar University, Assuit, Egypt; 8 Department of Physiology, College of Medicine, Jouf University, Sakaka, Saudi Arabia; 9 Department of Pharmacology, Faculty of Veterinary Medicine, Menoufia University, Shibin El Kom, Egypt

**Keywords:** Drug discovery, Medicinal biochemistry, Natural products, Parasitic diseases

## Abstract

**Background:**

The antiprotozoal and antioxidant activities of *Viola tricolor* and *Laurus nobilis* have been reported recently. Thus, the existing study pursued to assess the growth inhibition effect of methanolic extract of *V. tricolor* (MEVT) and acetonic extract of *L. nobilis* (AELN) against five *Babesia* parasites and *Theileria equi* in vitro and in vivo.

**Results:**

MEVT and AELN suppressed *Babesia bovis*, *B. bigemina*, *B. divergens*, *B. caballi*, and *T. equi* growth at half-maximal inhibitory concentration (IC_50_) values of 75.7 ± 2.6, 43.3 ± 1.8, 67.6 ± 2.8**,** 48 ± 3.8, 54 ± 2.1 μg/mL, and 86.6 ± 8.2, 33.3 ± 5.1, 62.2 ± 3.3, 34.5 ± 7.5 and 82.2 ± 9.3 μg/mL, respectively. Qualitative phytochemical estimation revealed that both extracts containing multiple bioactive constituents and significant amounts of flavonoids and phenols. The toxicity assay revealed that MEVT and AELN affected the mouse embryonic fibroblast (NIH/3 T3) and Madin–Darby bovine kidney (MDBK) cell viability with half-maximum effective concentrations (EC_50_) of 930 ± 29.9, 1260 ± 18.9 μg/mL, and 573.7 ± 12.4, 831 ± 19.9 μg/mL, respectively, while human foreskin fibroblasts (HFF) cell viability was not influenced even at 1500 μg/mL. The in vivo experiment revealed that the oral administration of MEVT and AELN prohibited *B. microti* multiplication in mice by 35.1 and 56.1%, respectively.

**Conclusions:**

These analyses indicate the prospects of MEVT and AELN as good candidates for isolating new anti-protozoal compounds which could assist in the development of new drug molecules with new drug targets.

## Background

Piroplasms, the causative factors of piroplasmosis, are among the most prevalent blood parasites in the world and therefore show a significant economic, medical and veterinary impact all over the world [1, 2]. The problems of parasite resistance, as well as the toxic residues to most of the commercially available antipiroplasmic drugs (diminazene aceturate (DMA), atovaquone (ATV), clindamycin, imidocarb dipropionate, azithromycin, and quinine) severely weaken their effective curative and protective approaches [3, 4]. Therefore, it is clear that the development of treatment options for piroplasmosis is vital for improving disease treatment and control.

Up to date, medicinal plants have been documented as an important source for discovering new pharmaceutical molecules that have been used to treat serious diseases [3, 5, 6]. Strikingly, previous reports stated that natural products and their derived compounds exhibit lesser side effects and improved efficacy than other synthetic counterparts [7]. Many plant species have been reported to have pharmacological activities attributable to their phytoconstituents such as glycosides, saponins, flavonoids, steroids, tannins, alkaloids, terpenes and accordingly.

*Viola tricolor* L., Sp. Pl. 935 (1753) (*V. tricolor*) (*Mnemion tricolor* (L.) Spach (1836), China violet, family Violaceae) is a genus of the flowering plants that have been used traditionally to treat many diseases such as bruises, fever, cough, flu, ulcers, malaria, and cancer [8–11]. Furthermore, its edible flowers have been reported for their importance to human health because of their richness in biologically active compounds [12–14]. It is worth noting that, 35 compounds have been previously reported for *V. tricolor* namely: 17 aliphatics, 8 sesquiterpenes, 4 monoterpenes, and 6 shikimic acid derivatives, in addition to the presence of bisabolol oxide A and B, bisabolene oxide and *trans*-β-farnesene volatile components [15, 16]. Moreover, *V. tricolor* contains 164 cyclotides, a plant cyclopeptide that is distinguished by cyclic cysteine-knot (CCK) motif, which occur naturally in many plants and possess numerous bioactivities including anthelmintic, molluscicidal, insecticidal, antimicrobial, hemolytic, anti-HIV, cytotoxic, immunosuppressive, and trypsin inhibitory activities [13, 17–20]. Moon et al. [21] studied the antimalarial effect of *Viola* petroleum ether extracts and its active component epi-oleanolic acid toward *Plasmodium falciparum* chloroquine-resistant *FcB1* strain. The *Viola* extracts showed high growth inhibition against *Leishmania donovani*, *Trypanosoma cruzi* and *P. falciparum* with IC_50_ values of 0.40, 1.86 and 2.76 μg/mL, respectively [5].

*Laurus nobilis* L., Sp. Pl. 369 (1753) (*L. nobilis*) (*Laurus undulata* Miller (1768)) is one of the most well-known herbs of the Lauraceae family that is also known as Bay or laurel leaves, and are frequently used in the traditional health care system [7, 22, 23]. Phytochemical studies on Bay leaves and its fruits have indicated various secondary metabolites including alkaloids, flavonols (kaempferol, myricetin, and quercetin), flavones (apigenin and luteolin), glycosylated flavonoids, sesquiterpene lactones, monoterpene, and germacrene alcohols. The main components isolated from *L. nobilis* were eugenol, elemicin, methyl eugenol, and α-terpinyl acetate [24]. Moreover, the phytochemical reports showed that leaves of *L. nobilis* possess sabinene, linalool, and 1, 8-cineole [25–27]. Traditionally, *L. nobilis* leaves have been used to treat gastrointestinal symptoms, such as eructation, epigastric bloating, impaired digestion and flatulence [28–30]. Fidan et al. [31] examined and proved the antimicrobial effects of the alcoholic extracts of *L. nobilis* and its essential oil. Ozcan et al. [6] documented the effectiveness of *L. nobilis* extracts in the treatment of rheumatoid arthritis and indigestion, as an antiseptic, a diaphoretic, and a diuretic. Moreover, their antioxidant property has been associated with the existence of methyl eugenol, elemicin and eugenol [24, 32].

Although *V. tricolor* and *L. nobilis* have been reported for several medicinal values, there is no evidence on their antipiroplasmic activity. Thus, the current study examined the effectiveness of methanolic extract of *V. tricolor* (MEVT) and acetonic extract of *L. nobilis* (AELN) on the multiplication of *T. equi*, *B. bigemina*, *B. bovis*, *B. caballi* and *B. divergens* using the in vitro fluorescence assay and their chemotherapy prospects against *B. microti*-infected mice.

## Methods

### Ethical statement

The in vivo experiments were performed in conformity with the local guidelines for animal experiments, as approved by the Obihiro University of Agriculture and Veterinary Medicine, Japan (accession number of the animal experiment: 28–111-2/28–110). This ethical approval was developed through the basic guidelines for the proper conduct of animal experimentation and related activities in Academic Research Institutions, Ministry of Education, Culture, Sports and Technology (MEXT), Japan.

### The chemical reagents

Stock solutions (100 mg (crude extract) / 1 mL (DMSO) and 10 mM) in dimethyl sulfoxide (DMSO) of crude extract and DMA (Ciba-Geigy Japan limited, Tokyo, Japan) and ATV (Sigma-Aldrich, Japan), respectively were stored at − 30 °C and used for antibabesiosis evaluation. Reference drugs (DMA and ATV) were used either singly and/or in combination with the two extracts for both the in vivo and in vitro experiments. For the fluorescence assay, SYBR Green I (SGI) stain (10,000×, Lonza, USA) was mixed with the lysis buffer containing saponin (0.016% w/v), EDTA (10 mM), Triton X–100 (1.6% v/v), and Tris (130 mM at pH 7.5) which was filtered using a polyethersulfone (0.22 μm) and kept at 4 °C.

### Herbal plants

*V. tricolor* flower and *L. nobilis* leaves were gathered from Delta, North part of Egypt from June 2016 to August 2016 and identified by the members of the Pharmacology and Chemotherapeutics Department, Faculty of Veterinary Medicine, Damanhour University, Egypt. *V. tricolor* and *L. nobilis* voucher specimen numbers are A0177103 (DPV) and A0177104 (DPV), respectively. An electric dryer (Sanyo Electric Co., Ltd., Osaka, Japan) was used to dry the plants at a temperature of 30 °C, then ground using a 60–80 mm mesh to a fine powder. Subsequently, fine plant powder (100 g) was extracted using methanol (99.8%) (Wako pure chemical Industrial, Ltd., Osaka, Japan) or acetone (99.5%) (Nacalai Tesque, Kyoto, Japan) (50 mL) and incubated for 72 h at a temperature of 30 °C. The preparation of slurry extract was performed following the method as previously described [3, 33, 34] and the extracted stock (100 mg / 1 mL DMSO) was stored at − 30 °C and used for antibabesial evaluation. The obtained extracts of the MEVT and AELN weight were 7.09 and 7.25 g, respectively, and the yield percentage was measured using the following formula [35]:

$$ \mathrm{Percentage}\ \mathrm{yield}\ \mathrm{of}\ \mathrm{extract}\mathrm{s}=\frac{\mathrm{Weight}\ \mathrm{of}\ \mathrm{the}\ \mathrm{obtained}\ \mathrm{extract}\ \mathrm{material}}{\mathrm{Weight}\ \mathrm{of}\ \mathrm{original}\ \mathrm{fine}\ \mathrm{plant}\ \mathrm{powder}\ \mathrm{used}}\times 100 $$

### Phytochemical examination of plant extracts

MEVT and AELN were examined for the existence of terpenoids, saponins, tannins, and alkaloids using several qualitative tests as previously reported elsewhere [36].

### Determination of total phenolic material

The total phenolic material concentration present in MEVT and AELN was detected using Folin-Ciocalteu (FC) assay as described elsewhere [37]. A volume of 500 μL of both extracts (1 mg/mL) was added to 1.5 mL of 10% FC reagent and mixed for 5 min. After that, the reaction mixture was further incubated for an additional 2 h after the addition of aliquot (3 mL) of 7.5% Na_2_CO_3_ solution. Finally, the absorbance was calculated at 760 nm and the total phenolic compounds were detected from a gallic acid standard curve and expressed as mg/g gallic acid equivalent (GAE) of the dry weight of the extract (mg GAE/g DW).

### Determination of total flavonoid material

Aluminium chloride (AlCl_3_) colorimetric assay was used for the examination of total flavonoid material in MEVT and AELN as previously determined [37]. Briefly, an aliquot (1 mL) of both extracts was added to 3 mL of solvent extracts, 3.8 mL of distilled water, 200 μL of 1 M potassium acetate and 200 μL of 10% AlCl_3_ and incubated for 30 min. The flavonoid content was detected from a catechin standard curve after measuring the absorbance at 420 nm and expressed as mg/g catechin equivalents of the dry weight of individual extract (mg CAE/g DW).

### Gas chromatography-mass spectrometry (GC-MS) analysis

The chemical composition of MEVT and AELN was performed using Trace GC-ISQ mass spectrometer (Thermo Scientific, Austin, TX, USA) with a direct capillary column TG–5MS (30 m × 0.25 mm × 0.25 μm film thickness) as previously described [38, 39]. The column oven temperature was initially held at 50 °C and then increased by 5 °C/min to 250 °C withhold 1 min then increased to 300 at the rate of 30 °C /min. The injector temperatures were kept at 260 °C. Helium was used as a carrier gas at a constant flow rate of 1 mL/min. The solvent delay was 4 min and diluted samples of 1 μL were injected automatically using an AS3000 Autosampler coupled with GC in the split mode. EI mass spectra were collected at 70 eV ionization voltages over the range of *m*/*z* 50–650 in full scan mode. The ion source and transfer line were set at 250 °C and 270 °C, respectively. The components were identified by comparison of their retention times and mass spectra with those of WILEY 09 and NIST 11 mass spectral databases.

### Parasites and mice

For the in vitro experiments, a German strain *B. divergens*, Argentine strain *B. bigemina*, Texas strain *B. bovis*, were cultivated in cattle RBCs, while USDA strains of equine piroplasm parasites (*T. equi* and *B. caballi*) were maintained in horse RBCs [34]. The parasite incubation occurred at 37 °C in a humidifying chamber under 90% N_2_, 5% O_2_, and 5% CO_2_ atmosphere using a microaerophilic stationary-phase culture. For conducting the in vivo study, female BALB/c mice, aged six-week, obtained from CLEA Japan were infected with *B. microti* Munich strain [1].

### In vitro cultivation of parasites

Roswell Park Memorial Institute (RPMI) 1640 medium (Sigma-Aldrich, Tokyo, Japan) replenished with 40% cattle serum and used as a growth medium for *B. divergens* parasite culture and culture medium 199 (M199) was used as a growth medium for *B. bigemina* and *B. bovis* replenished with 40% cattle serum. While *T. equi* was grown in M199 complemented with 40% horse serum containing hypoxanthine (MP Biomedicals, USA; final concentration 13.6 μg/mL). GIT medium replenished with 40% horse serum was used as a growth medium for *B. caballi* parasite culture. To ensure free-bacterial contamination, all medium was supplemented with amphotericin B (0.15 μg/mL) (Sigma-Aldrich, USA), streptomycin (60 U/mL) and penicillin G (60 U/mL).

### Assessment of the impacts of MEVT and AELN on RBCs of host

Prior to parasite subculture, 400 μg/mL of MEVT and AELN were mixed with fresh bovine and equine RBCs and incubated at a humidifying incubator for 3 h. Then *B. bovis* and *T. equi*-infected RBCs (iRBCs) were washed thrice with PBS and mixed with the pretreated-RBCs to achieve 1% parasitemia. Thereafter, using a 24-well plate, an aliquot of iRBCs (100 μL) was mixed with culture media (900 μL); the control RBCs were left untreated. To monitor the parasitemia and any side effects due to the pretreatment, Giemsa-stained smears were prepared every 24 h for 4 days.

### The inhibition assay of MEVT and AELN in vitro

The *Babesia* fluorescent assay was carried out on the in vitro culture as previously reported elsewhere [3]. Briefly, in three separate trials, using two-fold dilution, different concentrations MEVT, AELN, DMA, and ATV were prepared in the culture medium and added in 96-well plates in triplicate with 1% parasitemia for equine piroplasm parasites (*T. equi* and *B. caballi*) and *B. divergens* at 5% hematocrit (HCT), while for *B. bigemina* and *B. bovis* using 2.5% HCT. The positive control had iRBCs with final concentration of 0.2% of DMSO, whereas uninfected RBCs with medium served as the negative control. Afterward, parasite cultures were incubated for 4 consecutive days without changing medium at 37 °C humidifying incubator in 90% N_2_, 5% O_2_, and 5% CO_2_ atmosphere. On day four of culture, an aliquot (100 μL) of lysis buffer was added to 0.2 μL/mL SG1 per well; subsequently, it was covered with aluminum foil to prevent exposure to light. After a 6-h incubation at 37 °C, fluorescence readings were acquired on a spectrofluorimeter (Fluoroskan Ascent, Thermo Fisher Scientific, USA) with a 485 nm excitation wavelength and a 518 nm emission wavelength.

### Parasite viability test in vitro

The viability studies were monitored via microscopy as described elsewhere [1, 33]. Briefly, an aliquot (20 μL) of infected RBCs (1% parasitemia) was cultivated in 200 μL of media containing various concentrations of MEVT and AELN for 4 successive days, changing media daily. The concentrations used in this experiment were 0.25 ×, 0.5 ×, 1 ×, 2 ×, and 4 × the IC_50_. On the fifth day, a mixture of iRBCs (6 μL) from each well and fresh equine or bovine RBCs (14 μL) was transferred to another plate, cultured in a medium free from drug and then left for an additional 6 days. The total parasite clearance was recorded as negative, while the relapse of parasites was recorded as positive.

### Cell lines cultivation

Cultures of Human foreskin fibroblast (HFF; HFF-1 ATCC® SCRC-1041™), Madin–Darby bovine kidney (MDBK; ECACC) and mouse embryonic fibroblast (NIH/3 T3; ATCC® CRL-1658™) cells were retrieved from − 80 °C stock and cultured continuously at 37 °C under atmosphere 5% CO_2_ in our laboratory. The NIH/3 T3 and HFFs cells were preserved in Dulbecco Modified Eagle’s Medium (DMEM; Gibco, Grand Island, NY, USA), while MDBK cell line grown in Minimum Essential Medium Eagle (MEM; Gibco). All mediums were treated with 2 mM glutamine, 0.5% penicillin/streptomycin (Gibco) and 10% inactivated fetal bovine serum. Every 72 to 96 h, the medium was replaced, and once 80% confluence was reached, the cell collection was performed by sub-culture protocol. To confirm the absence of mycoplasma contamination, 4, 6-diamidino-2-phenylindole dihydrochloride (Sigma-Aldrich, USA) stain was used [3, 33].

### Cytotoxicity assay of MEVT and AELN on normal cells

The cell viability test was conducted in a 96-well plate as described elsewhere [2, 39]. Briefly, an aliquot of (100 μL) cells was implanted at 5 × 10^4^ cells/mL in DMEM or MEM with fetal bovine serum and incubated overnight under atmosphere 5% CO_2_ at 37 °C for attachment. Using two-fold dilutions, aliquots (10 μL) of herbal extracts and reference drugs were added in triplicate per well and further incubated for 24 h. The positive control wells containing cells mixed with the medium in 0.4% DMSO, whereas the negative control wells containing culture medium only. After a 24-h incubation, Cell Counting Kits-8 (CCK-8) (10 μL) was added per well and then plate incubation was conducted for an additional 3 h and a microplate reader was used to assess the absorbance at 450 nm.

### In vitro combination treatment of MEVT and AELN with DMA and ATV

Combination therapies of MEVT and AELN with DMA and ATV were tested using the fluorescence inhibition method as reported elsewhere [40, 41]. Five selected dilutions (0.25 ×, 0.5 ×, 1 ×, 2 × and 4 × the IC_50_) of the two herbal extracts with DMA and ATV were set up in three sets of duplicate wells and the parasite cultures were incubated for four consecutive days at 37 °C humidifying incubator in 90% N_2_, 5% O_2_, and 5% CO_2_ atmosphere without changing medium. The drug cultivation and the fluorescence values were detected after the addition of 2 × SGI mixed with lysis buffer to each well of the 96-well plate as described above. CompuSyn software was used for combination index (CI) values calculation and the synergetic degree was established as the average weighted CI values by using the following formulae; [(1 × IC_50_) + (2 × IC_75_) + (3 × IC_90_) + (4 × IC_95_)]/10 and the resulted values were demonstrated using the recommended CI scale; lower than 0.90 was considered synergetic, between 0.90–1.10 was considered additive, while higher than 1.10 was considered antagonistic developed previously [40, 41].

### Chemotherapeutic effects of MEVT and AELN against *B. microti*

MEVT and AELN were examined for its in vivo chemotherapeutic effectiveness using *B. microti*–infected BALB/c mice according to a procedure described elsewhere [1]. Twenty-five mice were placed in an environment free from pathogens with 22 °C temperature and adjusted humidity and under 12 h light and 12 h darkness and randomly distributed into five groups. The mice in groups 2 through 5 were obtained 500 μL of 1 × 10^7^*B. microti* iRBC by intraperitoneal (i.p.) injection. Group 1 served as a negative control and was neither infected nor treated. At 1% parasitemia, drug treatment of the mice by i.p. started, continuing for 5 days. Group 2 act as a positive control group and received 95% DDW and 5% DMSO. Group 3 was served as the reference drug control and received 25 mg kg^− 1^ body weight (BW) of DMA, while groups 4 and 5 obtained 150 mg kg^− 1^ BW of MEVT and AELN orally using oral gavage injection syringe, respectively. The drug administration lasted for 5 days starting from the fourth day to the eighth day post-infection (p.i.), and parasitemia was checked by preparing Giemsa-stained smears every 2 days in about 2000 RBCs by microscopy until day 32 post-infection (p.i.). Furthermore, the hematological parameters, including hemoglobin (HGB), RBCs, and HCT, were determined by an automatic hematology analyzer (Celltac α MEK-6450, Nihon Kohden, Japan) every 4 days. After finishing the in vivo experiment, an anesthetic system using an inhaler containing isoflurane was used to euthanize all mice by placing them in the induction chamber, adjusting the oxygen flowmeter to 0.8 to 1.5 L/min and vaporizer to 3 to 5%. When mice were completely anesthetized, all of them were killed by cervical dislocation according to the ethical approval confirmed by the Basic Guidelines for Proper Conduct of Animal Experiment and Related Activities in Academic Research Institutions, the Ministry of Education, Culture, Sports and Technology (MEXT), Japan.

### Data analysis

The IC_50_ values of the two extracts, DMA and ATV were established from the in vitro growth inhibition by nonlinear regression curve fit on a GraphPad Prism (GraphPad Software Inc., USA). While for in vivo*,* the significant variations (*p* < 0.05) among group mean values on parasitemia and one-way ANOVA Tukey’s test in GraphPad Prism version 5.0 was used to analyze hematology profiles in mice infected with *B. microti* [3].

## Results

### Plant extraction

The yield percentage of the MEVT and AELN were 7.09 and 7.25% w/w dry matter and dark in color.

### The inhibition assay of MEVT and AELN in vitro

**Fig. 1:**
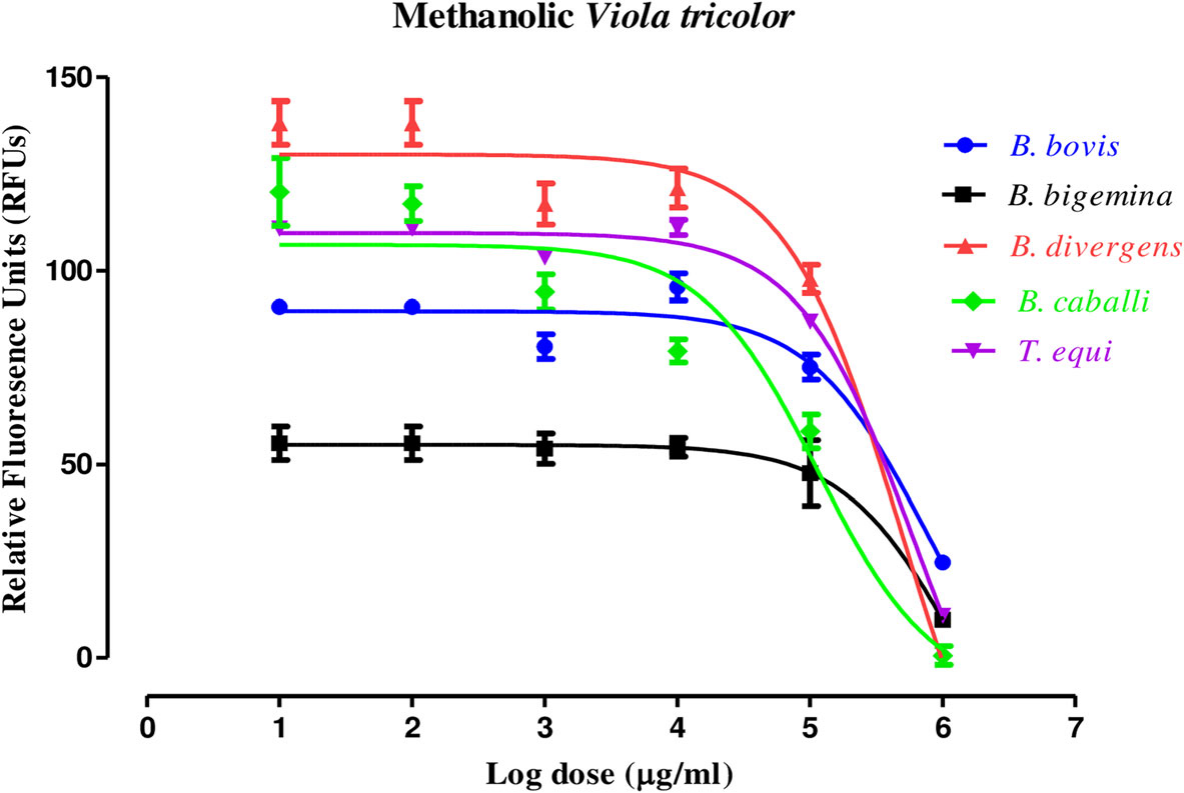
The relationship between the relative fluorescence units (RFUs) and the log concentrations of methanolic *V. tricolor* (MEVT) (μg/mL) on *T. equi*, *B. divergens*, *B. bigemina*, *B. caballi*, and *B. bovis*. The non-linear regression (curve fit analysis) in GraphPad Prism software (GraphPad Software Inc. USA) used for IC_50_’s calculation. The percentage of parasite growth inhibitory efficacy is calculated as the percentage of parasites inhibited divided by that of the positive control wells and the result was subtracted from the negative control wells

**Fig. 2:**
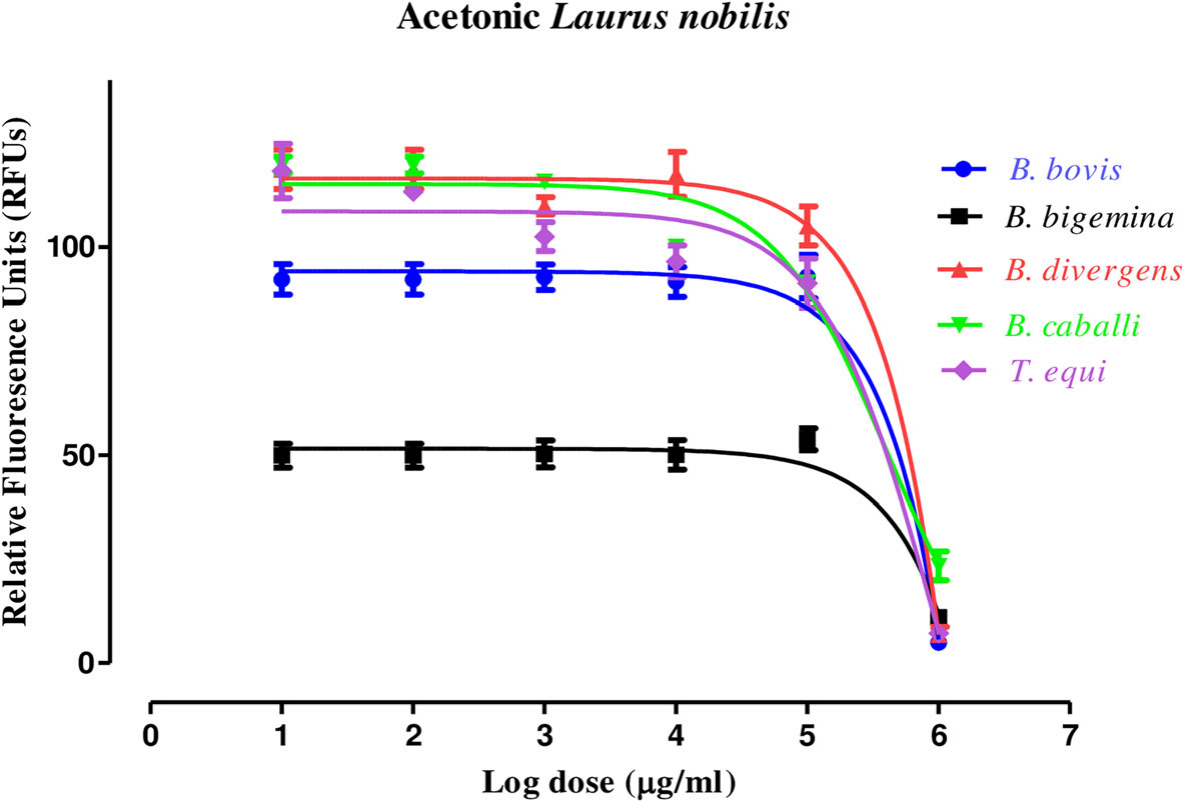
The relationship between the relative fluorescence units (RFUs) and the log concentrations of acetonic *L. nobilis* (AELN) (μg/mL) on *T. equi*, *B. divergens*, *B. bigemina*, *B. caballi*, and *B. bovis*. The non-linear regression (curve fit analysis) in GraphPad Prism software (GraphPad Software Inc. USA) used for IC_50_’s calculation. The percentage of parasite growth inhibitory efficacy is calculated as the percentage of parasites inhibited divided by that of the positive control wells and the result was subtracted from the negative control wells

**Fig. 3:**
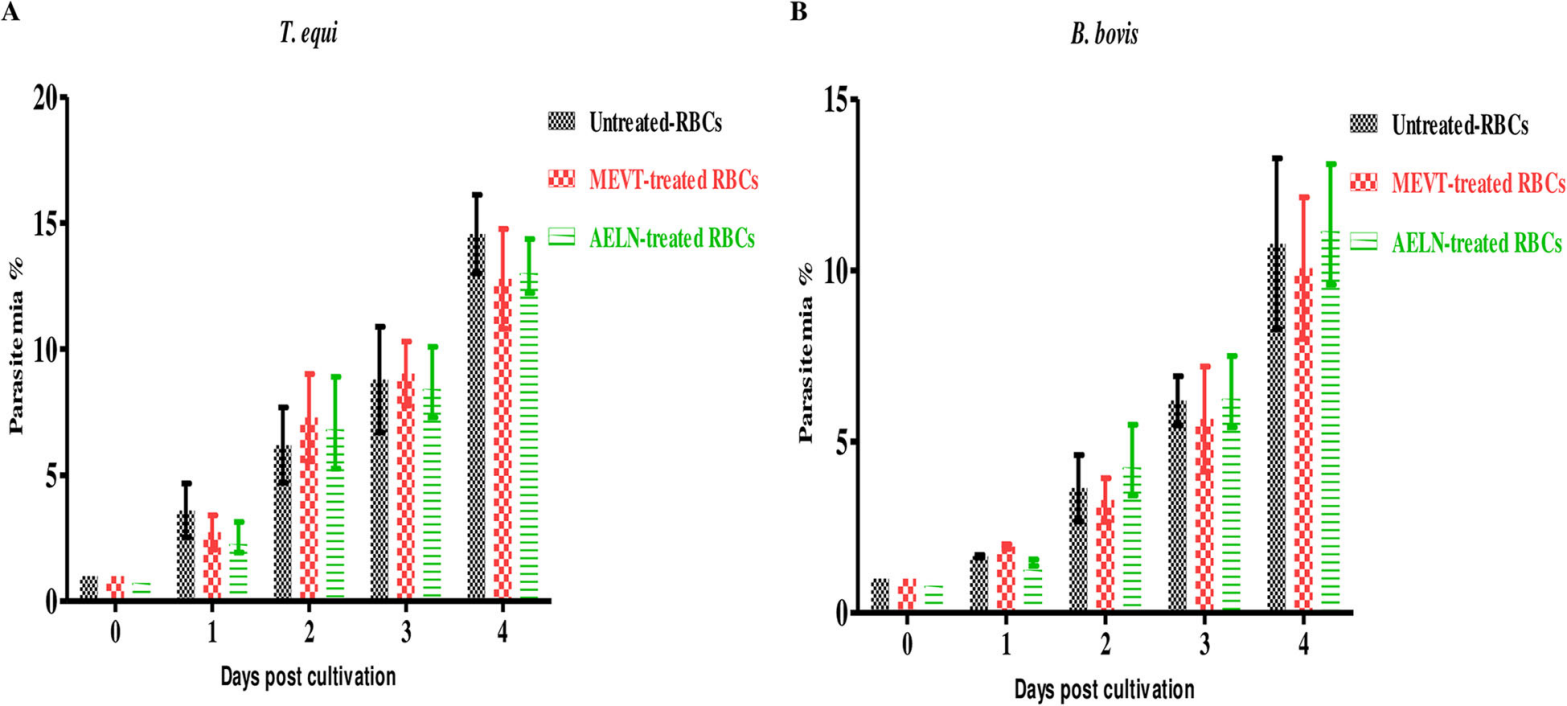
The growth of *B. bovis* and *T. equi* on RBCs pretreated with methanolic *V. tricolor* and acetonic *L. nobilis* in vitro. The curves showing the growth of *B. bovis* (**a**) and *T. equi* (**b**) on RBCs pretreated with 400 μg/mL of MEVT and AELN. The result was determined by counting using a microscope. The values obtained from three separate trials were used to determine the significance

MEVT (Fig. 1) and AELN (Fig. 2) significantly restricted (*p* < 0.05) *T. equi*, *B. divergens*, *B. caballi*, *B. bovis*, and *B. bigemina* multiplication in a dose-related manner. MEVT and AELN suppressed *T. equi*, *B. divergens*, *B. caballi*, *B. bigemina*, and *B. bovis* multiplication at IC_50_ values shown in Table 1. For the reference antibabesial drugs, DMA and ATV suppressed *T. equi*, *B. divergens*, *B. caballi*, *B. bigemina*, and *B. bovis* multiplication at IC_50_ values shown in Additional file 1: Table S1. The preparatory assessment of MEVT and AELN was carried out to detect their effect on the cattle and horse RBCs. The parasite proliferation did not show a significant difference between RBCs treated with MEVT or AELN and the untreated one for *B. bovis* and *T. equi* (Fig. 3a and b).

**Table 1:** IC_50_ and selective index values of methanolic *Viola tricolor* and acetonic *Laurus nobilis* extracts

Crude extract	*Babesia* and *Theileria*	IC_50_ (μg/mL)^a^	EC_50_ (μg/mL)^b^			Selective indices ^c^		
			MDBK	NIH/3 T3	HFF	MDBK	NIH/3 T3	HFF
MEVT	***B. bovis***	75.7 ± 2.6	930 ± 29.9	1260 ± 18.9	>1500	12.3	16.7	> 19.8
	***B. bigemina***	43.3 ± 1.8	930 ± 29.9	1260 ± 18.9	>1500	21.5	29.1	> 34.6
	***B. divergens***	67.6 ± 2.8	930 ± 29.9	1260 ± 18.9	>1500	13.8	18.6	> 22.2
	***B. caballi***	48 ± 3.8	930 ± 29.9	1260 ± 18.9	>1500	19.4	26.3	> 31.3
	***T. equi***	54 ± 2.1	930 ± 29.9	1260 ± 18.9	>1500	17.2	23.3	> 27.8
AELN	***B. bovis***	86.6 ± 8.2	573.7 ± 12.4	831 ± 19.9	>1500	6.6	9.6	> 17.3
	***B. bigemina***	33.3 ± 5.1	573.7 ± 12.4	831 ± 19.9	>1500	17.2	25	> 45.1
	***B. divergens***	62.2 ± 3.3	573.7 ± 12.4	831 ± 19.9	>1500	9.2	13.4	> 24.1
	***B. caballi***	34.5 ± 7.5	573.7 ± 12.4	831 ± 19.9	>1500	16.6	24.1	> 43.5
	***T. equi***	82.2 ± 9.3	573.7 ± 12.4	831 ± 19.9	>1500	7	10.1	> 18.2

^a^IC_50_ values of MEVT and AELN on all tested parasites in vitro. ^b^EC_50_ values of MEVT and AELN on the tested cell lines. The dose-response curve using nonlinear regression (curve fit analysis) was used to detect all of these values. The values obtained from the means of triplicate experiments. ^c^Selective index calculated as the ratio between the concentration that causes safety problems in cell lines and the concentration that is used for efficacy in each parasite

### Phytochemical evaluation of MEVT and AELN extracts

The primary examination of MEVT and AELN pointed to the existence of different phytoconstituents such as tannins, saponins, terpenoids and alkaloids that may be the main cause of their pharmaceutical properties (Table 2).

**Table 2:** Phytochemical screening of methanolic *Viola tricolor* and acetonic *Laurus nobilis* extracts

Extracts	Qualitative tests			
	Tannin	Saponin	Alkaloids	Terpenoid
Methanolic *Viola tricolor*	**+**	**+**	**+**	**+**
Acetonic *Laurus nobilis*	**+**	**+**	**+**	**+**

Results expressed as the mean values from three separate trials ± SD, + = Positive, − = Negative

### Establishment of total phenolic and flavonoid contents in MEVT and AELN

Considerable amounts of phenolic and flavonoid contents were observed in MEVT and AELN. Notably, AELN (71.2 ± 2.5 mg of GAE/g DW) showed the highest total phenolic content followed by MEVT (66.5 ± 2.9 mg of GAE/g DW) (Table 3). Moreover, AELN (33. 8 ± 9.7 mg of CAE/g DW) had the highest total flavonoid content followed by MEVT (39.2 ± 7.4 mg of CAE/g DW) (Table 3).

**Table 3:** Determination of total phenolic and flavonoid content of methanolic *Viola tricolor* and acetonic *Laurus nobilis* extracts

Extracts	Quantitative tests	
	Flavonoid mg CAE/g DW	Phenolic mg GAE/g DW
Methanolic *Viola tricolor*	33. 8 ± 9.7	66.5 ± 2.9
Acetonic *Laurus nobilis*	39.2 ± 7.4	71.2 ± 2.5

Results expressed as the mean values from three separate trials ± SD, mg GAE/g DW = gallic acid equivalents per gram of dry weight of extract, mg CAE/g DW = catechin equivalents per gram of dry weight of extract

### Gas chromatography-mass spectrometry (GC-MS) analysis

**Fig. 4:**
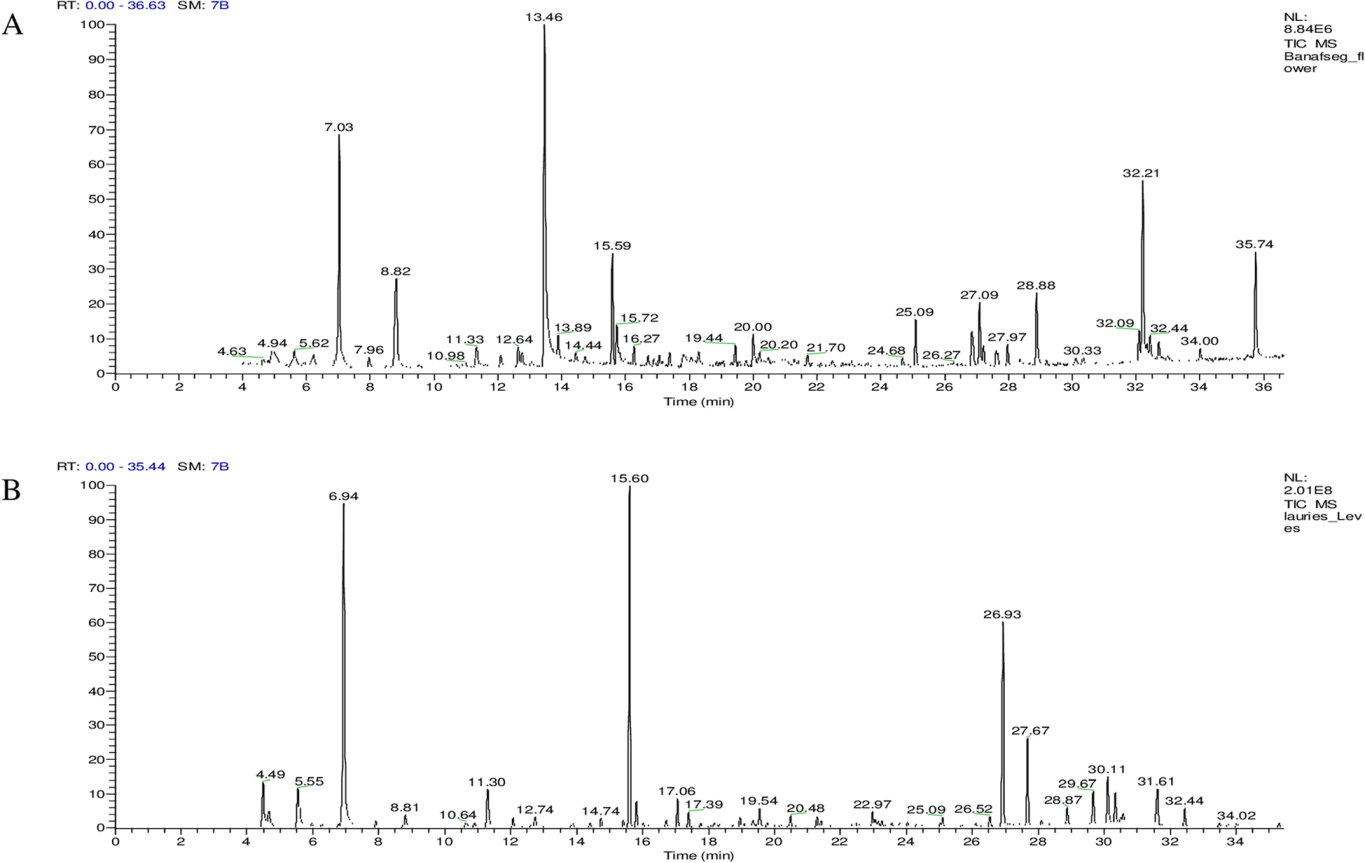
Gas chromatography-mass spectrometry analysis in the methanolic extract of *Viola tricolor* (**a**) and acetonic extract of *Laurus nobilis* (**b**)

The GC-MS analysis of MEVT and AELN revealed the existence of 27 and 20 phytochemical compounds, respectively. The identified chemical composition of MEVT is shown in Table 4 and represented 29 compounds. While the identified chemical composition of AELN is shown in Table 5 and represented 24 compounds. The phytochemical compounds’ identification was established on the basis of the peak area, and retention time. The active principles with their retention time (RT) and percentage of peak area (%) are expressed in Fig. 4a and b.

**Table 4:** The chemical composition of MEVT by GC-MS

Peak	R.t^a^	Name	Area %	Molecular Weight	Molecular formula
1	4.93	Methanesulfonylacetic acid	1.99	138	C_3_H_6_O_4_S
2	6.62	(+)-Sabinene	1.34	136	C_10_H_16_
3	7.03	Eucalyptol	10.35	154	C_10_H_18_O
4	8.82	Undecane	7.36	156	C_11_H_24_
5	11.33	L-à-Terpineol	1.42	154	C_10_H_18_O
6	12.09	Cinnamic acid, linalyl ester	0.74	284	C_19_H_24_O_2_
7	12.64	Benzaldehyde, 4-(1-methylethyl)-	1.10	148	C_10_H_12_O
8	12.76	(−)-Carvone	0.80	150	C_10_H_14_O
9	13.46	Cinnamaldehyde, (E)-	20.23	132	C_9_H_8_O
10	13.89	Anethole	1.30	148	C_10_H_12_O
11	15.59	à-Terpinyl acetate	5.47	196	C_12_H_20_O_2_
12	15.72	Benzene, 1-Methoxy-2-nitro-	2.74	152	C_7_H_7_NO_3_
13	16.27	Copaene	2	204	C_15_H_24_
14	16.70	ç-Elemene	0.66	204	C_15_H_24_
15	17.06	Methyleugenol	0.58	178	C_11_H_14_O_2_
16	17.38	Caryophyllene	0.80	204	C_15_H_24_
17	18.29	1,2-Benzenedicarboxylic acid, dimethyl ester	1.40	194	C_10_H_10_O_4_
18	19.44	à-Muurolene	1.19	204	C_15_H_24_
19	20.00	Cadina-1(10),4-diene	1.96	204	C_15_H_24_
20	25.09	2H-Pyran-3-ol, tetrahydro-2,2,6-trimethyl-6-(4-methyl-3-cyclohexen-1-yl)-, [3S-[3à,6à(R^a^)]]-	2.45	238	C_15_H_26_O_2_
21	26.85	Isopropyl myristate	2.92	270	C_17_H_34_O_2_
22	27.09	Neophytadiene	5.94	278	C_20_H_38_
23	27.21	2-Pentadecanone, 6,10,14-trimethyl-	1.07	268	C_18_H_36_O
24	28.88	Hexadecanoic acid, methyl ester	4.16	270	C_17_H_34_O_2_
25	32.09	12,15-Octadecadienoic acid, methyl ester	1.50	294	C_19_H_34_O_2_
26	32.21	1-Hexadecanol, 2-methyl-	9.94	256	C_17_H_36_O
27	32.44	Phytol	0.95	296	C_20_H_40_O
28	32.71	Heptadecanoic acid, 9-methyl-, methyl ester	1.12	298	C_19_H_38_O_2_
29	35.74	Docosane	6.11	310	C_22_H_46_

^a^R.t, retention time (min)

**Table 5:** The chemical composition of AELN by GC-MS

Peak	R.t^a^	Name	Area %	Molecular Weight	Molecular formula
1	4.49	Cumene	3.19	120	C_9_H_12_
2	4.67	à-Pinene	1.12	136	C_10_H_16_
3	5.54	(+)-Sabinene	3.46	136	C_10_H_16_
4	6.94	Eucalyptol	21.68	154	C_10_H_18_O
5	8.81	3,7-Dimethylocta-1,6-dien-3-ol	1.05	154	C_10_H_18_O
6	11.30	Beta-fenchol	3.22	154	C_10_H_18_O
7	12.07	Terpineol	1.75	154	C_10_H_18_O
8	12.74	Carvone	0.86	150	C_10_H_14_O
9	15.60	Terpinyl acetate	20.21	196	C_12_H_20_O_2_
10	15.81	Eugenol	1.60	164	C_10_H_12_O_2_
11	17.06	Methyleugenol	1.70	178	C_11_H_14_O_2_
12	17.39	Caryophyllene	0.95	204	C_15_H_24_
13	19.54	Germacra-1(10),4(15),5-triene	1.82	204	C_15_H_24_
14	20.48	cis-à-Bisabolene	0.79	204	C_15_H_24_
15	21.29	(−)-Spathulenol	0.70	220	C_15_H_24_O
16	21.41	Caryophyllene oxide	0.56	220	C_15_H_24_O
17	22.97	Eudesm-4(14)-en-11-ol	0.81	222	C_15_H_26_O
18	26.93	2(3H)-Benzofuranone, 6-ethenylhexahydro-6-methyl-3-methylene-7-(1-methylethenyl)-, [3aS (3aà,6à,7á,7aá)]-	13.87	232	C_15_H_20_O_2_
19	27.67	Gazaniolide	7.56	230	C_15_H_18_O
20	29.67	Spirafolide	2.30	246	C_15_H_18_O_3_
21	30.11	Dehydrocostuslactone	3.28	230	C_15_H_18_O_2_
22	30.33	Eremanthin	2.01	230	C_15_H_18_O_2_
23	31.61	Costunolide	2.99	232	C_15_H_20_O_2_
24	32.44	Phytol	1.29	296	C_20_H_40_O

^a^R.t, retention time (min)

### Viability assay

The viability test revealed that MEVT at 4 × IC_50_ (173.2 and 192 μg/mL) completely suppressed *B. bigemina* and *B. caballi*, respectively, while 6 × IC_50_ (324, 405.6 and 454.2 μg/mL) concentration cleared *T. equi*, *B. divergens* and *B. bovis*, respectively (Table 4). AELN-treated *B. bigemina, B. divergens, T. equi,* and *B. bovis* completely suppressed at 6 × IC_50_ (199.8, 373.2, 493.2 and 519.6 μg/mL), respectively except *B. caballi* completely cleared at 2 × IC_50_ (69 μg/mL) concentration (Table 6).

**Table 6:** Viability of parasites treated with MEVT and AELN

Extracts	Conc.	Parasites				
		*B. bovis*	*B. bigemina*	*B. divergens*	*B. caballi*	*T. equi*
MEVT	**0.25 × IC**_**50**_	**+**	**+**	**+**	**+**	**+**
	**0.5 × IC**_**50**_	**+**	**+**	**+**	**+**	**+**
	**1 × IC**_**50**_	**+**	**+**	**+**	**+**	**+**
	**2 × IC**_**50**_	**+**	**+**	**+**	**+**	**+**
	**4 × IC**_**50**_	**+**	**–**	**+**	**–**	**+**
	**6 × IC**_**50**_	**–**	**–**	**–**	**–**	**–**
AELN	**0.25 × IC**_**50**_	**+**	**+**	**+**	**+**	**+**
	**0.5 × IC**_**50**_	**+**	**+**	**+**	**+**	**+**
	**1 × IC**_**50**_	**+**	**+**	**+**	**+**	**+**
	**2 × IC**_**50**_	**+**	**+**	**+**	**–**	**+**
	**4 × IC**_**50**_	**+**	**+**	**+**	**–**	**+**
	**6 × IC**_**50**_	**–**	**–**	**–**	**–**	**–**
	**Untreated control**	**+**	**+**	**+**	**+**	**+**

Results are calculated as the mean values from three separate trials ± SD, a positive (+) indicates parasites regrowth, and a negative (−) shows the parasites total clearance after drug pressure withdrawal using microscopy assay

### Toxicity activities of MEVT and AELN

The toxicity assay of MEVT and AELN revealed that MEVT and AELN affected MDBK and NIH/3 T3 cell viability at EC_50_ of 930 ± 29.9, 1260 ± 18.9 and 573.7 ± 12.4, 831 ± 19.9 μg/mL, respectively. While HFF cell viability was not affected by the two extracts even at the highest concentration used at 1500 μg/mL. The highest selectivity index (ratio of the effective concentration of the two extracts on the cell cultures to their inhibition concentration on the parasites) of MEVT was achieved on *B. bigemina* and found to be 21.5, 29.1 and > 34.6 times toward MDBK, NIH/3 T3, and HFF cells, respectively, while the lowest was achieved on *B. bovis* and found to be 12.3, 16.7 and > 19.8 times toward MDBK, NIH/3 T3, and HFF cells, respectively. For AELN, the highest selectivity index was 17.2, 25 and > 45.1 times toward *B. bigemina* versus MDBK, NIH/3 T3, and HFF cells, respectively, while the lowest were 6.6, 9.6 and > 17.3 toward *B. bovis* versus MDBK, NIH/3 T3, and HFF cells, respectively (Table 1).

### Combination treatment in vitro

The MEVT/DMA combined treatment was additive toward *T. equi*, *B. divergens*, *B. bovis* and *B. caballi*, while showed an antagonistic efficacy toward *B. bigemina*. AELN/DMA combined treatment was antagonistic toward *T. equi*, *B. divergens*, *B. bigemina*, and *B. bovis*, while was additive toward *B. caballi* (Table 7). The combination effects of MEVT and AELN with ATV were shown in Table 5.

**Table 7:** Combination effect of MEVT and AELN with DMA and ATV

Parasite	Drug combination	CI value	Degree of association
*B. bovis*	MEVT + **DMA**	1.200	**Additive**
	AELN + **DMA**	5.742	**Antagonism**
	MEVT + **ATV**	0.209	**Synergism**
	AELN + **ATV**	0.822	**Synergism**
*B. bigemina*	MEVT + **DMA**	4.991	**Antagonism**
	AELN + **DMA**	3.266	**Antagonism**
	MEVT + **ATV**	0.273	**Synergism**
	AELN + **ATV**	0.776	**Synergism**
*B. divergens*	MEVT + **DMA**	1.072	**Additive**
	AELN + **DMA**	8.667	**Antagonism**
	MEVT + **ATV**	0.304	**Synergism**
	AELN + **ATV**	1.003	**Additive**
*B. caballi*	MEVT + **DMA**	0.989	**Additive**
	AELN + **DMA**	0.947	**Additive**
	MEVT + **ATV**	0.291	**Synergism**
	AELN + **ATV**	0.392	**Synergism**
*T. equi*	MEVT + **DMA**	0.893	**Additive**
	AELN + **DMA**	8.965	**Antagonism**
	MEVT + **ATV**	0.844	**Synergism**
	AELN + **ATV**	0.999	**Additive**

*MEVT* methanolic extract of *V. tricolor*, *AELN* acetonic extract of *L. nobilis*, *DMA* diminazene aceturate, *ATV* atovaquone. CI denotes the combination index value

### The in vivo chemotherapeutic potential of MEVT and AELN in mice

**Fig. 5:**
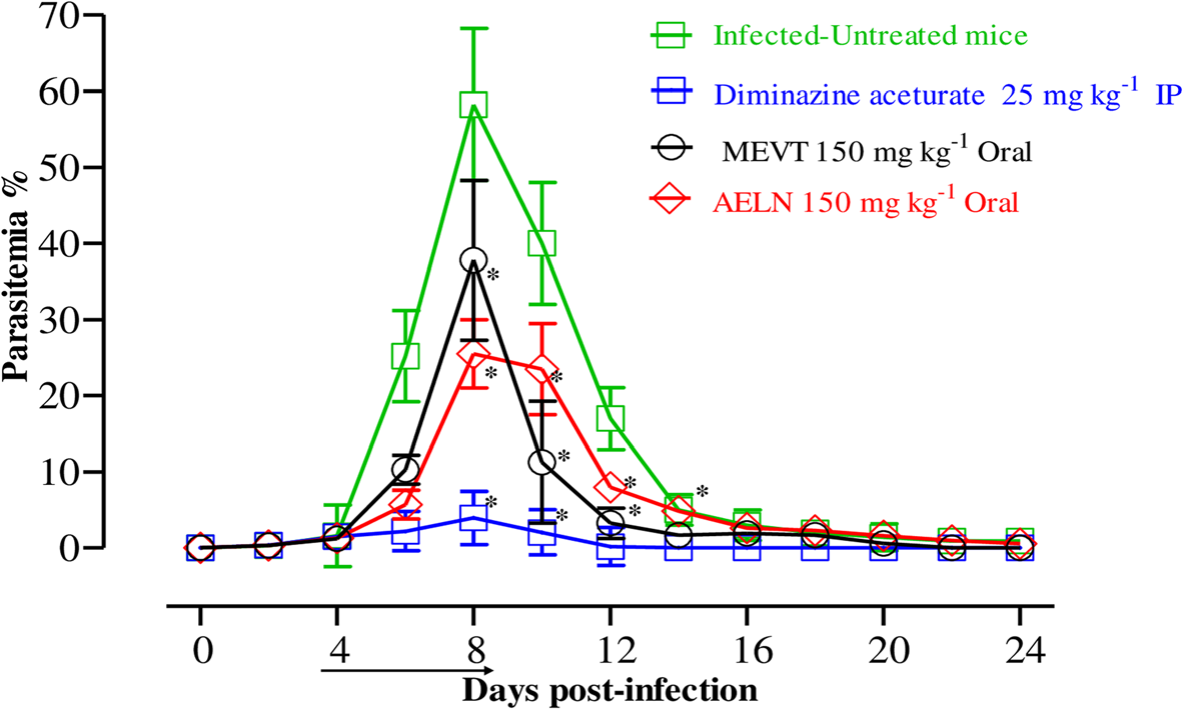
The growth inhibition of methanolic *V. tricolor* and acetonic *L. nobilis* on *B. microti* in vivo*.* Inhibitory effect of MEVT and AELN on the growth of *B. microti* in mice, based on observations taken from five mice per experimental group. The arrow indicates 5 consecutive days of treatment. Asterisks indicate statistically significant (*p* < 0.05) differences of parasitemia between treated groups and the untreated control group based on one-way ANOVA Tukey’s test using GraphPad Prism version 5.0 for Windows (GraphPad Software Inc., San Diego, CA, USA). Parasitemia was calculated by counting iRBCs among 2000 RBCs using Giemsa-stained thin blood smears. The data were the mean and standard deviation from two separate experiments

**Fig. 6:**
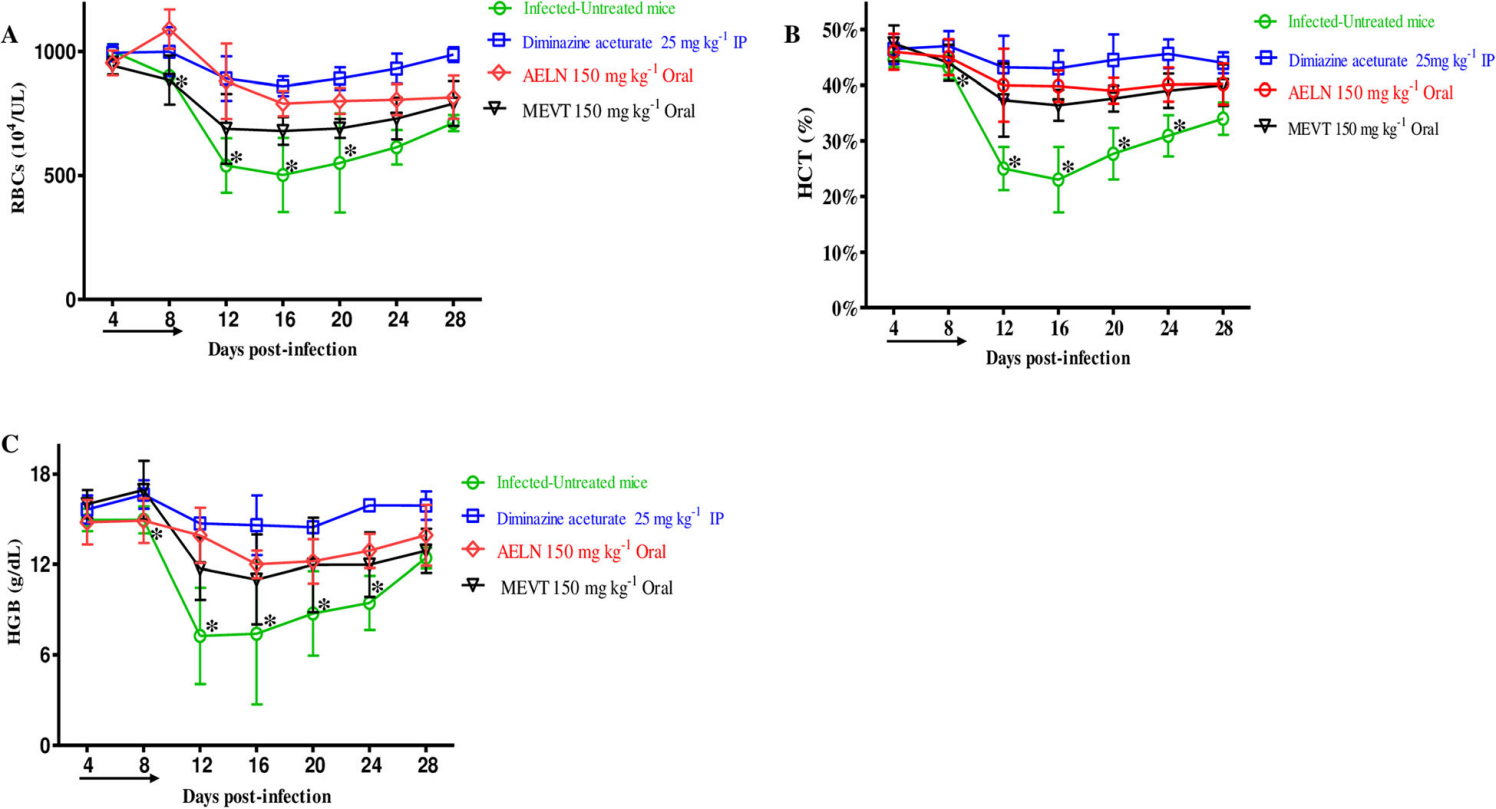
The hematology parameters changes in methanolic *V. tricolor* (MEVT) and acetonic *L. nobilis* (AELN)-treated mice in vivo. Graphs showing the changes in hematocrit count (**a**), hemoglobin percentage (**b**), and red blood cell count (**c**) in mice treated with DMA and MEVT and AELN. The arrow indicates 5 consecutive days of treatment. Asterisks indicate statistical significance (*p < 0.05*) based on one-way ANOVA Tukey’s test using GraphPad Prism version 5.0 for Windows (GraphPad Software Inc., San Diego, CA, USA). The data were the mean and standard deviation from two separate experiments (five mice per group)

To investigate the chemotherapeutic potential of MEVT and AELN in vivo*,* six-female BALB/c mice were infected by *B. microti* and the two extracts were administered for 5 days after the infection reach 1% parasitemia. On eighth day post-infection (p.i), the control group treated with double distillate water (DDW) exhibited rapid growth of parasitemia reached 58.2% and the parasitemia reduced slowly on the subsequent days. The level of parasitemia in all treated groups reached 37.8, 25.5 and 3.9% in MEVT (150 mg kg^− 1^), AELN (150 mg kg^− 1^), and DMA (25 mg kg^− 1^), respectively, at 8 days p.i (Fig. 5). Additionally, the hematology parameters; HCT percentage, RBCs count and HGB concentration (Fig. 6a-c) showed a significant difference in the MEVT- and AELN-treated groups when compared with the positive control group. Whereas, the comparison of the hematology parameters during in vivo studies showed no significant difference (*p <* 0.05) between MEVT-, AELN-treated groups as compared to the DMA-treated group.

## Discussion

Plant extracts possess significant therapeutic effects with minimal side effects for the treatment of many infectious diseases [33], thus making medicinal plants an attractive choice for the source of new therapeutic compounds [34]. In spite of the antiparasitic effect of *V. tricolor* and *L. nobilis*, they have not been evaluated against piroplasmosis. Therefore, this study investigates the in vitro as well as in vivo antipiroplasmic efficacy of MEVT and AELN.

The existing study revealed that MEVT and AELN possess various biologically active compounds and the primary screening emphasized that both extracts contain terpenoids, alkaloids, flavonoids, and tannins (Table 2). The qualitative examination revealed the presence of significant amounts of polyphenols and flavonoids (Table 3). Notably, this finding conforms to the report by Chandra et al. [42] and Alejo-Armijo et al. [43] who revealed the existence of these active constituents in both extracts. It has been shown that all these secondary metabolites have many therapeutic properties and are known to be pharmacologically active components.

The phytochemical constituents of MEVT and AELN were detected using GC-MS analyses. The analysis revealed that MEVT consisted of 29 compounds and the main chemical components identified were cinnamaldehyde, (E) - (20.23%), eucalyptol (10.35%), 1-hexadecanol, 2-methyl- (9.94%), undecane (7.36%), docosane (6.11%), Cadina-1(10), neophytadiene (5.94%) and à-terpinyl acetate (5.47%). While AELN was found to possess 24 compounds and the main chemical components identified were eucalyptol (21.68%), terpinyl acetate (20.21%), 2(3H)-benzofuranone, 6-ethenylhexahydro-6-methyl-3-methylene-7-(1-methylethenyl)-, [3aS (3aà,6à,7á,7aá)] (13.87%), and gazaniolide (7.56%). Some of our GC-MS results were consistent with previous reports [25, 26, 44], however, the difference in some compounds may be attributed to the type of solvents used and the extraction method.

The in vitro experiments indicated that MEVT and AELN suppressed the in vitro multiplication of piroplasm parasites. Previous reports documented that *V. tricolor* and *L. nobilis* herbal extracts have potent efficacy toward *Plasmodium*, *Trypanosoma*, and *Leishmania* [5, 21]. For instance, Dua et al. [5] exhibited the antiplasmodial activity of petroleum ether extract of *Viola canescens* with an IC_50_ equal to 2.76 μg/mL, as well as its efficacy toward *Leishmania donovani* and *Trypanosoma cruzi* with IC_50_ values of 0.40 μg/mL and 1.86 μg/mL, respectively. In addition to antiprotozoal effectiveness *V. tricolor* and *L. nobilis* extracts, Ozcan et al. [6] and Koike et al. [14] reported that *L. nobilis* and *V. tricolor* has strong antioxidant, antimicrobial and antibacterial activity. Interestingly, recent studies documented the antiprotozoal activities of various bioactive molecules identified in our GC-MS analysis. For instance, Castaño Osorio and Giraldo García [45]. reported that different sesquiterpene lactones and costunolide, isolated from *Laurus nobilis,* showed antiprotozoal efficacy against *Trypanosoma brucei rhodesiense*. Colares et al. [46] and Le et al. [47] proved the antileishmanial activity of eugenol and methyleugenol against promastigotes of *L. amazonensis*. Moreover, Charma et al. [48] documented the antileishmanial effect of cinnamaldehyde and eugenol with IC_50_ values of 0.681 and 1.426 g/ml, respectively. Eucalyptol and caryophyllene showed antitrypanosomal and antileishmanial activities, respectively [49]. Therefore, we hypothesized that eucalyptol, caryophyllene, cinnamaldehyde, eugenol, methyleugenol, and costunolide are the main active compounds responsible for the antipiroplasmic activity of MEVT and AELN.

The CCK test used to examine the cytotoxicity of MEVT and AELN revealed their effect on NIH/3 T3, HFFs, and MDBK cell viability with a slightly high selective index value. Meaning that MEVT and AELN are more likely to affect the viability of piroplasm parasites rather than host cells. Notably, this finding conforms to the report by Dua et al. [5], who revealed that *Viola* extract exhibited antiplasmodial activity with some cytotoxic efficacy toward the L-6 cell line. Furthermore, Kivçak and Mert [28]. documented the safety of water and ethanol *L. nobilis* extracts on the Brine shrimp. Previous reports documented the cytotoxic activities of the *L. nobilis* extracts and its main component 1, 8-cineole in a human neuroblastoma cell line (SH-SY5Y), as well as, the cytotoxic activity of the *V. tricolor* extracts and its main component cyclotides were evaluated in normal cell lines. Their EC_50_ values were higher than 400 μg/mL concentration, suggesting their safety on the normal cell lines [17, 23].

Nowadays, combination chemotherapies are being reported to alleviate serious diseases, including pulmonary tuberculosis, malignancy, immune deficiency syndrome, and some protozoal diseases to promote higher therapeutic efficacy. The MEVT-DMA or ATV combined effects were additive toward all tested parasites. Likewise, AELN combined with DMA and ATV showed additive and synergistic effects toward *T. equi*, *B. caballi*, *B. bigemina*, *B. divergens* and *B. bovis*. These results are compatible with Batiha et al. [3] who previously investigated the in vitro combined efficacy of methanolic extracts of *S. aromaticum* and *C. sinensis* with DMA and ATV toward piroplasm parasites. They concluded that these combined effects are property for developing new chemotherapy techniques against piroplasm parasites*.* Together, these findings in our study emphasize that these combinations have prospects to be used as a treatment option of animal and human babesiosis.

In the in vivo experiment, the oral administration of MEVT and AELN resulted in 35.1 and 56.1% suppression in the parasitemia level on the eighth-day p.i., respectively when compared with 93.2% restriction showed by DMA. The effectiveness of MEVT and AELN was comparable to that shown by Batiha et al. [3], who reported that the methanolic extracts of *S. aromaticum* and *C. sinensis* on the eighth-day p.i. resulted in 69.2 and 42.4% inhibition in the parasitemia at day 8 p.i., respectively. Interestingly, no obvious toxic signs were observed in MEVT- and AELN-treated mice.

Nevertheless, MEVT and AELN, like DMA, prohibited anemia development in mice, although temporal reductions were observed in HCT, RBCs, and HGB. Furthermore, neither the MEVT nor the AELN treatments showed any apparent toxic symptoms or promoted anemia in uninfected mice. Interestingly, many reports have shown remarkable antioxidant and pro-oxidative effects of ethanol and water extract of the whole plant of *Viola* by prohibiting the reactive oxygen species (ROS) generation or stimulating the protein detoxification [11]. Additionally, Emam et al. [22] observed the significant antioxidant, antipyretic, and anti-inflammatory properties after *L. nobilis* extracts treatment. Moreover, Ozcan et al. [6] reported that essential oil, methanolic extract of seed oil and seed oil from *L. nobilis* possess antioxidant and antimicrobial activities*.* Such medicinal characteristics are important for piroplasmosis treatment because piroplasmosis infection is not only correlated with emaciation and poor growth performance in cattle but also with immunosuppression and overproduction of reactive oxygen and nitrogen species [3]. The limitation of this study is performing the cytotoxicity assay in vitro using cell lines, and it is recommended to evaluate the cytotoxic activity of our extracts in vivo.

These findings emphasize the MEVT and AELN ability to eradicate *B. microti* in mice. Taken together, these findings support that MEVT and AELN could be a potential source of alternate chemotherapy against *B. microti* infection in humans.

## Conclusions

To our knowledge, this is the first antipiroplasmic evaluation of methanolic *V. tricolor* and acetonic *L. nobilis* extracts against piroplasm parasites*.* MEVT and AELN exhibited an in vitro growth inhibitory effect against five piroplasm species as well as chemotherapeutic efficacy toward *B. microti* in vivo*.* Furthermore, the combination treatment of our herbal extracts with DMA and ATV demonstrated synergistic and additive effectiveness against all testes parasites. Our GC-MS analysis results documented the existence of several phytochemical molecules that may be responsible for the babesicidal activities of MEVT and AELN. Therefore, it is recommended to evaluate the antipiroplasmic efficacy of the GC-MS identified compounds for the future discovery of a novel potential drug against piroplasmosis. And evaluate the actual mode of action employed against the recovery of piroplasm parasites.

## Supplementary information

**Additional file 1: Table S1.** The IC_50_ and selective indexes value of ATV and DMA.

**Additional file 2.** ARRIVE guideline checklist.

## Data Availability

All data generated or analyzed during this study are included in this published article [and its Additional files 1 and 2].

## Abbreviations

AELN: Acetonic extract of *L. nobilis*
ATV: Atovaquone
CCK-8: Cell Counting Kit-8
CI: Combination index
DDW: Double distilled water
DMA: Diminazene aceturate
DMEM: Dulbecco’s Modified Eagle’s Medium
DMSO: Dimethyl sulfoxide
EC_50_: Half maximum effective concentrations
EDTA: Ethylenediaminetetraacetic acid
HCT: Hematocrit
HFF: Human foreskin fibroblast
HGB: Hemoglobin
IC_50_: Half maximal inhibitory concentration
iRBCs: infected red blood cells
M199: Medium 199
MDBK: Madin-Darby bovine kidney
MEM: Minimum Essential Medium Eagle
MEVT: Methanolic extract of *V. tricolor*
NIH/3 T3: Mouse embryonic fibroblast
RBCs: Red blood cells
