# Supplementary material for: Gas chromatography-mass spectrometry analysis, phytochemical screening and antiprotozoal effects of the methanolic Viola tricolor and acetonic Laurus nobilis extracts
Source: BMC Complement Med Ther. 2020 Mar 17;20:87. doi: 10.1186/s12906-020-2848-2 (PMC7077018; doi:10.1186/s12906-020-2848-2)
Supplement: Supplementary file 1 — Additional file 1: Table S1. The IC50 and selective indexes value of ATV and DMA. [file 12906_2020_2848_MOESM1_ESM.docx]

**Table S1** IC_50_ and selectivity index of atovaquone and diminazene aceturate

| **Control drug** | **Parasites** | **IC_50_ (µg/mL)^a^** | **EC_50_ (µg/mL)^b^ cell lines** | | | **Selective indices^c^** | | |
| --- | --- | --- | --- | --- | --- | --- | --- | --- |
|  |  |  | **MDBK** | **NIH/3T3** | **HFF** | **MDBK** | **NIH/3T3** | **HFF** |
| **ATV** | ***B. bovis*** | 0.015 ± 0.001 | ˃ 100 | ˃ 100 | ˃ 100 | ˃ 6666.7 | ˃ 6666.7 | ˃ 6666.7 |
|  | ***B. bigemina*** | 0.26 ± 0.03 |  |  |  | ˃ 384.6 | ˃ 384.6 | ˃ 384.6 |
|  | ***B. divergens*** | 0.014 ± 0.002 |  |  |  | ˃ 7142.9 | ˃ 7142.9 | ˃ 7142.9 |
|  | ***B. caballi*** | 0.038 ± 0.01 |  |  |  | ˃ 2631.6 | ˃ 2631.6 | ˃ 2631.6 |
|  | ***T. equi*** | 0.035 ± 0.01 |  |  |  | ˃ 2857.1 | ˃ 2857.1 | ˃ 2857.1 |
| **DMA** | ***B. bovis*** | 0.25 ± 0.02 | ˃ 100 | ˃ 100 | ˃ 100 | ˃ 400 | ˃ 400 | ˃ 400 |
|  | ***B. bigemina*** | 0.11 ± 0.01 |  |  |  | ˃ 909 | ˃ 909 | ˃ 909 |
|  | ***B. divergens*** | 0.35 ± 0.03 |  |  |  | ˃ 285.7 | ˃ 285.7 | ˃ 285.7 |
|  | ***B. caballi*** | 0.003 ± 0.001 |  |  |  | ˃ 33333.3 | ˃ 33333.3 | ˃ 33333.3 |
|  | ***T. equi*** | 0.37 ± 0.01 |  |  |  | ˃ 270.3 | ˃ 270.3 | ˃ 270.3 |

a IC_50_ values of ATV and DMA on all tested parasites in vitro. b EC_50_ values of ATV and DMA on the tested cell lines. The dose-response curve using nonlinear regression (curve fit analysis) was used to detect all of these values. The values obtained from the means of triplicate experiments. c Selective index calculated as the ratio between the concentration that causes safety problems in cell lines and the concentration that is used for efficacy in each parasite
