# Supplementary material for: Gas chromatography-mass spectrometry analysis, phytochemical screening and antiprotozoal effects of the methanolic Viola tricolor and acetonic Laurus nobilis extracts
Source: BMC Complement Med Ther. 2020 Mar 17;20:87. doi: 10.1186/s12906-020-2848-2 (PMC7077018; doi:10.1186/s12906-020-2848-2)
Supplement: Supplementary file 2 — Additional file 2. ARRIVE guideline checklist. [file 12906_2020_2848_MOESM2_ESM.docx]

|  | ITEM | RECOMMENDATION |
| --- | --- | --- |
| Title | Page 1 | Anti-piroplasmic potential of the methanolic *Viola tricolor* and acetonic *Laurus nobilis* extracts |
| Abstract | Page 2 and 3 | **Background:** The absence of the effective chemotherapeutic agents and the plenteous resistance to almost all antipiroplasmic drugs have accentuated the urgent need for new chemotherapeutics and drug targets for both prophylaxis and chemotherapy. The antiprotozoal and antioxidant activities of *Viola tricolor* and *Laurus nobilis* have been reported recently.  **Objective of the study:** to evaluate the *in vitro* and *in vivo* antibabesial activity of methanolic extract of *V. tricolor* (MEVT) and acetonic extract of *L.* *nobilis* (AELN) alone and combined with diminazene aceturate (DA) and atovaquone (AQ) were investigated.  **Methods:** The fluorescence-based assay was used for evaluating the growth inhibition effects of MEVT and AELN on four *Babesia* species and *Theileria equi in vitro*, and on the multiplication of *B. microti* in BALB/c mice. The cytotoxicity assay was tested on Madin-Darby bovine kidney (MDBK), mouse embryonic fibroblast (NIH/3T3) and human foreskin fibroblast (HFF) cell lines.  **Results:** MEVT and AELN inhibited the growth of *Babesia bovis*, *B. bigemina*, *B. divergens, B. caballi*, and *T. equi* at half-maximal inhibitory concentration (IC_50_) values of 75.7 ± 2.6, 43.3 ± 1.8, 67.6 ±2.8**,** 48 ± 3.8, 54 ± 2.1 µg/mL, and 86.6 ± 8.2, 33.3 ± 5.1, 62.2 ± 3.3, 34.5 ± 7.5 and 82.2 ± 9.3 µg/mL, respectively. The toxicity assay showed that MEVT and AELN affected the viability of Madin–Darby bovine kidney (MDBK) and mouse embryonic fibroblast (NIH/3T3) cell lines with half-maximum effective concentrations (EC_50_) of 930 ± 29.9, 1260 ± 18.9 µg/mL, and 573.7 ± 12.4, 831 ± 19.9 µg/mL, respectively, while human foreskin fibroblasts (HFF) cell lines viability was not affected even at 1500 µg/mL. In the *in vivo* experiment, oral treatments with 150 mg/kg of MEVT and AELN inhibited the growth of *Babesia microti* in mice by 35.1% and 56.1%, respectively.  **Conclusions:** These analyses suggest that MEVT and AELN are good candidates for isolation of anti-protozoal compounds which could serve as new lead structures for drug development. |
| INTRODUCTION |  |  |
| Background | Paragraphs 1 and 2, page 4  Paragraph 4, page 5  Paragraph 5, page 6 | Piroplasms, the causative factors of piroplasmosis, are some of the most ubiquitous and widespread blood parasites in the world and consequently, they have considerable worldwide economic, medical and veterinary impact (Tayebwa et al., 2018). The problems of parasite resistance as well as the toxic residues to most of the commercially available antipiroplasmic drugs (diminazene aceturate (DA) and imidocarb dipropionate, clindamycin, quinine, atovaquone (AQ) and azithromycin) seriously weaken efficient therapeutic and prophylactic control approaches (Batiha et al., 2019a; Beshbishy et al., 2019a). There is an urgent and continuing need to identify alternative drugs to treat piroplasmosis.  Medicinal plants are the best friends of human being dedicating to humanity without selfishness. Medicinal plants have been a good source of new pharmacological molecules. For example, natural products could be a potential alternative for controlling the pathogen associated with diseases (Dua et al., 2011; Batiha et al., 2019b). Natural products and their derivatives represent more than 50 % of the drugs in clinical use in the world (Patrakar et al., 2012). One of the paramount reasons for pursuing natural products chemistry resides in the actual or potential pharmacological activity to be found in alkaloids, terpenoids, coumarins, flavonoids, lignans and glycosides.  *Viola tricolor* (China violet) is a genus of the flowering plants in the violet family Violaceae (Feyzabadi et al., 2017). Medicinal properties for all parts of these plants have been reported, including anti-Plasmodial, anti-HIV, anthelmintic, insecticidal, molluscicidal, antimicrobial, uterotonic, cytotoxic, hemolytic, trypsin inhibitory, and immunosuppressive activities (Chen et al., 2005; Svangard et al., 2004; Toiu et al., 2007; Porrini et al., 2011).  *Laurus nobilis* one of the most well-known plants from the Lauraceae family, which is also known as Bay or laurel leaves, most frequently used in the traditional health care system (Patrakar et al., 2012; Emam et al., 2010; Pacifico et al., 2013). A variety of biologically active phytochemicals of *L. nobilis*  used in herbal medicines as antimicrobial, antiinflammatory, antipyretic and in the treatment of gastrointestinal problems, such as epigastric bloating, impaired digestion, eructation, and flatulence rheumatoid arthritis and indigestion, as an antiseptic, a diaphoretic, and a diuretic (Simic et al., 2003; Ozcan et al., 2010; Kivçak et al., 2002; Kaurinovic et al., 2010). |
| Objectives | Introduction,paragraph 6, page 7 | This study aimed to: 1) evaluate the inhibitory effect of extract of *V. tricolor* (MEVT) and acetonic extract of *L.* *nobilis* (AELN) against the growth of bovine *Babesia* (*B. bovis*, *B. bigemina* and *B. divergens*), and equine piroplasm parasites (*B. caballi* and *T. equi*) using *in vitro* culture. 2) Testing the effect of combination treatments of MEVT and AELN combined with DA and AQ against the growth of *B. bovis*, *B. bigemina*, *B. divergens*, *B. caballi*, and *T. equi in vitro*.  3) Evaluate the two extracts on rodent *Babesia* that infects humans (*B. microti*) using a BALB/c mouse model. |
| METHODS |  |  |
| Ethical statement | Paragraph 1, Page 13 | The experiments described in this study were conducted according to the rules of care description and animal use in research published by Obihiro University of Agriculture and Veterinary Medicine, Japan. The protocol was approved by the Animal Experimentation Ethics committee at Obihiro University of Agriculture and Veterinary Medicine (accession number of animal experiment: 28-111-2/28-110). These regulations were established by Fundamental Guidelines for Proper Conduct of Animal Experiment and Related Activities in Academic Research Institutions, the Ministry of Education, Culture, Sports and Technology (MEXT), Japan. |
| Study design | Paragraph 12, Page 19 | 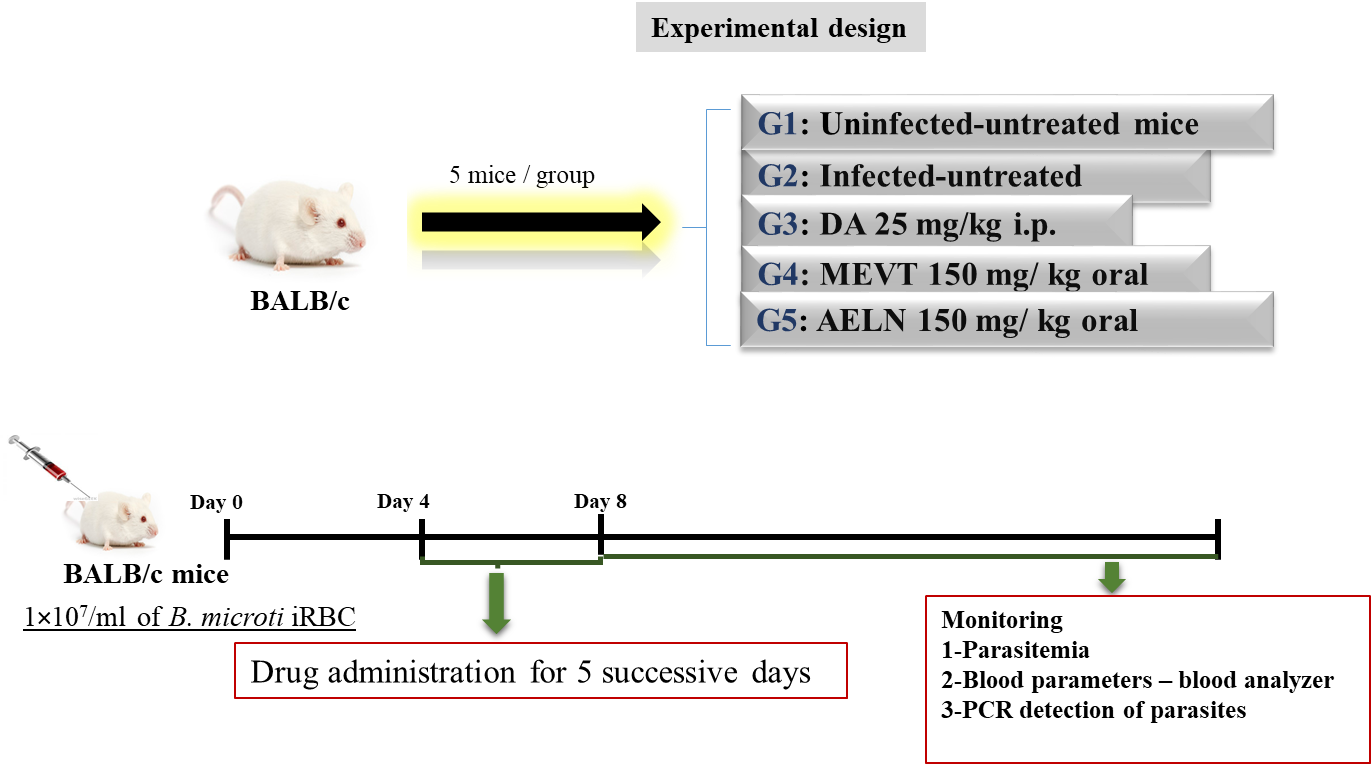Twenty-five 6-weeks-old female BALB/c mice were housed in cages and divided equally into five groups. Four groups were injected intraperitoneally with 1 × 10^7^ *B. microti* iRBCs, while the fifth group was left uninfected to act as the negative control. When the average parasitemia in all infected mice reached approximately 1%, the 5-day drug treatment was initiated. |
| Experimental procedures | Paragraph 12, page 20 | In 6-weeks-old female BALB/c mice, the growth inhibition of MEVT and AELN against *B. microti* was evaluated as previous described by (Batiha et al., 2019a). Twenty-five mice were housed under a pathogen-free environment with controlled temperature (22℃) and humidity and a 12 h light/ dark cycle and divided equally into five groups. Four groups were injected intraperitoneally with 1 × 10^7^ *B. microti* iRBCs, while the fifth group was left uninfected to act as the negative control. When the average parasitemia in all infected mice reached approximately 1%, the 5-day drug treatment was initiated. MEVT and AELN were administrated orally at a dose of 150 mg kg^-1^ to the first and second groups respectively, DA (reference antibabesial drug) was administrated intraperitoneally to the mice in the third group at a dose of 25 mg kg^-1^. The fourth group was administered with double distilled water (DDW) as the vehicle used. After the *in vivo* inhibition assay, all mice were euthanized using an inhalation anesthesia system containing isoflurane by placing mice in the induction chamber, adjusting the oxygen flowmeter to 0.8 to 1.5 L/min and vaporizer to 3% to 5%. When mice were completely anesthetized, all of them were killed by cervical dislocation according to the ethical approval established by Fundamental Guidelines for Proper Conduct of Animal Experiment and Related Activities in Academic Research Institutions, the Ministry of Education, Culture, Sports and Technology (MEXT), Japan. |
| Experimental animals | Paragraph 4, page 14 | The Munich strain of *B. microti* was used to infect 6-week female BALB/c mice purchased from CLEA Japan (Tokyo, Japan) to conduct the *in vivo* experiments. |
| Housing and husbandry | Paragraph 12, page 19 | Twenty-five BALB/c mice were housed under a pathogen-free environment with controlled temperature (22℃) and humidity and a 12 h light/ dark cycle and divided equally into five groups. Each group contain five mice and housed in cages. |
| Sample size | Paragraph 12, page 19 | Twenty-five BALB/c mice were divided equally into five groups. Each group contain five mice. The experiment was repeated two times. |
| Allocating animals to experimental groups | Paragraph 12, page 19 | Twenty-five BALB/c mice were divided equally into five groups. Four of the groups were injected intraperitoneally with 1 × 10^7^ *B. microti* iRBCs, while one of the groups was left uninfected to act as the negative control. |
| Experimental outcomes | Paragraph 12, page 20 | The levels of parasitemia in all mice were detected daily by Giemsa-stained thin blood smears prepared from venous tail blood until 32 days post-infection every two days. The hematocrit (HCT), hemoglobin (HGB), and RBCs counts were determined by collecting 10 μL of blood from each mouse every 96 h and this was used to monitor the blood parameters by using the Celltac α MEK-6450 automatic hematology analyzer (Celltac α MEK-6450; Nihon Kohden, Tokyo, Japan). After the *in vivo* inhibition assay, all mice were euthanized using an inhalation anesthesia system containing isoflurane by placing mice in the induction chamber, adjusting the oxygen flowmeter to 0.8 to 1.5 L/min and vaporizer to 3% to 5%. When mice were completely anesthetized, all of them were killed by cervical dislocation according to the ethical approval established by Fundamental Guidelines for Proper Conduct of Animal Experiment and Related Activities in Academic Research Institutions, the Ministry of Education, Culture, Sports and Technology (MEXT), Japan. |
| Statistical methods | Paragraph 13, pages 20-21 | The non-linear regression (curve fit), available in GraphPadPrism (GraphPad Software Inc., USA) was used to calculate IC_50_ of extracts and DA from the percentage of inhibition. While for *in vivo,* the differences among groups regarding the parasitemia and hematology profiles were analyzed using one-way ANOVA Tukey's test using GraphPad Prism version 5.0. The difference was considered statistically significant if a *p* < 0.05 was obtained |
| RESULTS |  |  |
| Baseline data | Methods, Paragraph 4, page 19 | 6-weeks-old female BALB/c mice |
| Numbers analysed | Methods, Paragraphs 12 and 13 Pages 20 and 21 | Twenty-five mice were divided equally into five groups. Student’s *t*-test, available in the GraphPad Prism software. |
| Outcomes and estimation | Paragraph 6, pages 8 and 9 | Control group treated with double distillate water (DDW) exhibited rapid growth of parasitemia reached 58.2 % on day 8 post-infection (p.i) and the parasitemia decreased gradually on the following days. The peak parasitemia level in the treated groups reached to 37.8%, 25.5% and 3.9% in 150 mg kg^-1^ MEVT, 150 mg kg^-1^ AELN, and 25 mg kg^-1^ DA, respectively, at 8 days p.i (Fig. 4). Additionally, the hematology parameters; hematocrit (HCT) percentage, RBCs count and hemoglobin (HGB) concentration (Fig. 5a-c) showed a significant difference in the MEVT- and AELN-treated groups as compared to the infected-untreated group. |
| Adverse events | N/A | No apparent adverse effects of the extracts were observed. |
| DISCUSSION |  |  |
| Interpretation/ scientific implications | Paragraph 7, page 12 | The chemotherapeutic effects produced by MEVT and AELN implicate that these two extracts could be a potential source of alternative therapy against bovine babesiosis, equine piroplasmosis, and human babesiosis. |
| Generalizability/ translation | Conclusion, paragraph 1, page 12 | To our knowledge, this is the first antipiroplasmic evaluation of methanolic *V. tricolor* and acetonic *L. nobilis* extracts against *Babesia* and *Theileria* parasites*.* MEVT and AELN showed a growth inhibitory effect against several *Babesia* species and *T. equi in vitro* as well as *B. microti in vivo.* Furthermore, the combination treatment of our herbal extracts with DA, and AQ showed synergistic and additive effects against *Babesia* and *Theileria* parasites. These results suggest the antipiroplasmic effect of MEVT and AELN, however, further studies are required to isolate and identify bioactive ingredient(s) responsible for the therapeutic action against *Babesia* and *Theileria* parasites that could be used as a lead compounds in the search for effective and novel antipiroplasmic drugs. |
| Funding | Paragraph 1, page 23 | This study was supported by the Ministry of Higher Education Egypt, the Japanese Society for the Promotion of Science, and the Ministry of Education, Culture, Sports, Science and Technology, Japan (JSPS) (KAKEN Grant Number: 18H02337). |
